# Risk Evaluation for Acute Kidney Injury Induced by the Concomitant Use of Valacyclovir, Analgesics, and Renin–Angiotensin System Inhibitors: The Detection of Signals of Drug–Drug Interactions

**DOI:** 10.3389/fphar.2019.00874

**Published:** 2019-08-08

**Authors:** Ichiro Inaba, Yuki Kondo, Shinya Iwasaki, Satoko Tsuruhashi, Ayano Akaishi, Kazuya Morita, Kentaro Oniki, Junji Saruwatari, Yoichi Ishitsuka, Tetsumi Irie

**Affiliations:** ^1^Department of Clinical Chemistry and Informatics, Graduate School of Pharmaceutical Sciences, Kumamoto University, Kumamoto, Japan; ^2^Central Pharmacy Nagamine, Kumamoto, Japan; ^3^Division of Pharmacology and Therapeutics, Graduate School of Pharmaceutical Sciences, Kumamoto University, Kumamoto, Japan; ^4^Center for Clinical Pharmaceutical Sciences, Faculty of Pharmaceutical Sciences, Kumamoto University, Kumamoto, Japan

**Keywords:** valacyclovir, acute kidney injury, acetaminophen, nonsteroidal anti-inflammatory drugs, renin–angiotensin system inhibitors, drug–drug interaction

## Abstract

**Background:** Drug-related acute kidney disease is a common side effect of valacyclovir (VACV) treatment. Although analgesics are frequently administered concomitantly with VACV to treat the pain of herpes zoster, the differences between nonsteroidal anti-inflammatory drugs (NSAIDs) and acetaminophen in relation to VACV-related acute kidney injury (AKI) are unclear. The risk for AKI with concomitant use of VACV and renin–angiotensin system (RAS) inhibitors that can cause AKI *via* a similar mechanism to NSAIDs is also unknown. We therefore evaluated the association between concomitant use of these drugs and VACV-related AKI, which was characterized according to the Japanese Adverse Drug Event Report (JADER) database.

**Methods:** We analyzed data from the JADER database, which is a spontaneous reporting system. The reporting odds ratio was used to evaluate the signals of AKI.

**Results:** A high proportion of VACV-related AKI cases occurred in summer. There was an increase in AKI signal in cases with concomitant use of VACV and NSAIDs, while no increase was detected in cases with concomitant use of VACV and acetaminophen. AKI events in cases with concomitant use of VACV and NSAIDs were more frequent in older and female patients and those with hypertension. Additionally, a signal increase for VACV-related AKI was observed with concomitant use of RAS inhibitors, with or without NSAIDs.

**Conclusions:** We identified a seasonal variation in VACV-related AKI. Additionally, our findings indicate that acetaminophen might represent a safer analgesic than NSAIDs with respect to VACV-related AKI. We also identified candidate risk factors for AKI with concomitant use of NSAIDs, such as older age, female sex, and hypertension. Although further studies are warranted, our findings highlight the need to consider concomitant drug use and seasonal factors that lead to urinary output loss so that VACV-related AKI can be avoided.

## Introduction

Acute kidney injury (AKI) is a common and serious condition, with a prevalence of 15–60% among inpatients (Mehta et al., 2004; Hoste et al., 2015). AKI can also lead to longer hospitalization times and higher mortality (Chertow et al., 2005). In particular, the incidence of drug-related AKI may be as high as 60% (Shahrbaf, 2015).

Valacyclovir (VACV), an antiviral prodrug of acyclovir (ACV), is effective against herpes zoster-associated pain and skin lesions (Colin et al., 2000; Lin et al., 2001). VACV has largely replaced ACV for the treatment of herpes virus infections because it is more effective when administered orally (Asahi et al., 2009). Therefore, VACV is widely used for the treatment of herpes zoster and herpes simplex worldwide, including in Japan. However, VACV is known as a common cause of drug-related AKI (Sugimoto et al., 2008; Roberts et al., 2011; Zhang et al., 2016).

Analgesics such as nonsteroidal anti-inflammatory drugs (NSAIDs) are frequently administered alongside VACV to treat pain related to herpes zoster. However, some NSAIDs have been shown to be associated with increased risk of VACV-related AKI in the FDA spontaneous reporting system (Yue et al., 2014; Yue et al., 2018). Acetaminophen, an analgesic, is generally regarded as safer than NSAIDs with regard to kidney function (Griffin et al., 2000). To our knowledge, no study to date has compared the renal safety of NSAIDs and acetaminophen when used in combination with VACV. Furthermore, renin–angiotensin system (RAS) inhibitors, including renin inhibitors, angiotensin-converting enzyme inhibitors, and angiotensin receptor blockers, are known to cause AKI (Turgut et al., 2010). RAS inhibitors can reduce glomerular filtration rate (GFR) and urinary output, similar to NSAIDs (Pouwels et al., 2013). However, the risk of AKI with concomitant use of VACV and RAS inhibitors remains unknown. In the present study, we evaluated the AKI signal for concomitant use of VACV with analgesics and/or RAS inhibitors from the Japanese Adverse Drug Event Report (JADER) database. Additionally, we characterized VACV-related AKI from the JADER database.

## Methods

### Study Population

JADER is a Japanese database of adverse event reports submitted to the Pharmaceuticals and Medical Devices Agency (PMDA) and can be accessed from the PMDA website (https://www.pmda.go.jp/safety/info-services/drugs/adr-info/suspected-adr/0004.html). JADER consists of four data tables: “DEMO” (demography of each case); “DRUG” (information about drug administration); “REAC” (information about adverse events); and “HIST” (information about comorbidities). Our analysis was conducted in adverse event cases submitted to JADER between April 2004 and April 2018, which comprised 524,662 adverse drug event (ADE) cases. Cases with incomplete information on patient sex and age were excluded from the analysis. Patients aged less than 20 years were also excluded, as NSAIDs and RAS inhibitors are not commonly administered to children and the inclusion of these patients would have made it difficult to determine the relationship between age group and differences in concomitant medication use. Duplicate cases were merged, and a total of 440,818 ADE cases were subsequently reviewed for analysis.

### AKI Signal Detection

Adverse events and comorbidities were coded using the preferred terms (PTs) in the Medical Dictionary for Regulatory Activities (MedDRA). In the present study, MedDRA/J ver. 21.1, the Japanese version of MedDRA, was used. AKI events were identified using PTs related to AKI in the Standardised MedDRA Query (SMQ) for “acute renal failure” [20000003], as described in previous studies (Yue et al., 2014; Perlman et al., 2017). The PTs used for identification of AKI events are shown in **Supplementary Table 1a**.

Cases were grouped based on the use of target drugs (VACV, NSAIDs, acetaminophen, and RAS inhibitors). In this analysis, NSAIDs and RAS inhibitors were defined according to the drug names listed in Kyoto Encyclopedia of Genes and Genomes (KEGG) Drug database as “DGROUP: Nonsteroidal anti-inflammatory drug (NSAID)” (KEGG DGROUP: Nonsteroidal anti-inflammatory drug (NSAID)) and “DGROUP: Renin–angiotensin system inhibitor” (KEGG DGROUP: Renin–angiotensin system inhibitor). Cases with only topical use of NSAIDs were excluded. Aspirin was excluded as an NSAID in this study as it was typically administered as an antiplatelet drug at a low dose. Additionally, 3,080 cases with concomitant use of NSAIDs and acetaminophen (NSAIDs + acetaminophen cases and VACV + NSAIDs + acetaminophen cases, including seven VACV-related AKI cases) were excluded from the comparison analysis between NSAIDs and acetaminophen (**Table 2**) because the purpose of the analysis was a risk comparison between NSAIDs and acetaminophen, with concomitant use of VACV. As a result, a total of 437,738 cases (including the 1,212 VACV-related AKI cases) were included in the comparison analysis.

The reference group consisted of cases who used neither VACV nor the target candidate for drug–drug interactions (for example, the reference group in **Table 2** comprised cases in which VACV, NSAIDs, or acetaminophen were not used). Monotherapy of VACV, NSAIDs, and RAS inhibitors was defined as cases who received no other target drug in each analysis (for example, “VACV monotherapy cases” in **Figure 3** consisted of patients who used VACV but did not use NSAIDs or RAS inhibitors). Therefore, the number of cases of monotherapy differed between **Table 2** and **Figure 3**. To evaluate the signals of AKI, the reporting odds ratios (RORs) and 95% confidence intervals (CIs) for AKI were calculated using the following formula, with RORs adjusted for age, sex, and reporting year:

$$\mathrm{ROR}=\frac{a/c}{b/d},95\%\text{ CI}=\exp\left\{\log(\mathrm{ROR})\pm 1.96\sqrt{\frac{1}{a}+\frac{1}{b}+\frac{1}{c}+\frac{1}{d}}\right\}$$

where *a* represents cases that belonged to the group and identified as AKI; *b* represents cases that did not belong to the group and identified as AKI; *c* represents cases that belonged to the group and did not identify as AKI; and *d* represents cases that did not belong to the group and did not identify as AKI.

Adjusted RORs were calculated by logistic regression using age-stratified group, gender, and reporting year as covariates, according to a previous study (Ueda et al., 2015). To confirm the AKI signals, we calculated the proportional reporting ratio (PRR) (Evans et al., 2001) and empirical Bayes geometric mean (EBGM) (DuMouchel, 1999). The criteria for signal detection were as follows: PRR, AKI cases ≥ 3; PRR ≥ 2, χ^2^ ≥ 4; and EBGM, lower limit of the 95% CI (EB05) ≥ 2.

Signals of drug–drug interaction were examined in two steps. AKI signals were evaluated as positive when the lower limit of the 95% CI of the ROR exceeded 1. If the AKI signal was positive, the signals of drug–drug interactions were also defined as positive if the adjusted RORs of the cases with concomitant use were higher than those of other index groups, and the 95% confidence intervals were mutually exclusive as reported previously (Puijenbroek et al., 1999; Li et al., 2015).

### Characteristics of AKI With Concomitant Use of VACV and NSAIDs

To identify risk factors for AKI with concomitant use of VACV and NSAIDs, a case–control study of the cohort was conducted. We performed multivariate logistic regression analysis to evaluate the risk of AKI with concomitant use of VACV and NSAIDs. The outcome variable was AKI events identified using the PTs related to AKI in the SMQ “Acute renal failure” [20000003]. The explanatory variables were older age (≥70 years), sex, and various comorbidities associated with AKI. The selected comorbidities were as follows: chronic kidney disease (CKD) (Singh et al., 2010), hypertension (Hsu et al., 2008), diabetes mellitus (Mehran et al., 2004), and heart failure (Rosner and Okusa, 2006). These comorbidities were defined from the “HIST” table by SMQ (“chronic kidney disease” [20000213], “hypertension” [20000147], “hyperglycaemia/new onset diabetes mellitus” [20000041], and “cardiac failure” [20000004]). The PTs used for identification of comorbidities are shown in **Supplementary Tables 1b, 1c, 1d, and 1e**. The explanatory variables in the final model were selected using a stepwise method according to the *p* value. The concordance statistic (area under the receiver operating characteristic curve) of the final model was 0.75.

### Statistical Analysis

Multivariate analysis was performed using logistic regression analysis. The significance value was set at *p* < 0.05. All statistical analyses were performed using JMP^®^ Pro 14.0 software (SAS Institute Inc., Cary, NC, USA).

## Results

### Characteristics of VACV-Related AKI

A total of 1,219 AKI cases associated with VACV use were reported between April 2004 and April 2018, and the characteristics of these cases are shown in **Table 1**. Approximately 70% of the patients were female, and VACV-related AKI tended to increase with age and was particularly common among patients aged 70s and 80s. A high proportion (57.9%) of VACV-related AKI events occurred in the first week after administration.

**Table 1:** The characteristics of VACV-related AKI cases (*n* = 1,219).

Variable	Number (%)
**Sex**	
Male	342 (28.1)
Female	877 (71.9)
**Age**	
20s	7 (0.6)
30s	6 (0.5)
40s	15 (1.2)
50s	57 (4.7)
60s	152 (12.5)
70s	433 (35.5)
80s	444 (36.4)
90s	104 (8.5)
100s	1 (0.1)
**Duration of treatment until AKI**	
Within 7 days	706 (57.9)
8–14 days	30 (2.5)
15–28 days	8 (0.7)
Over 29 days	16 (1.3)
Unknown	459 (37.7)
**Outcome**	
Recovery	605 (49.6)
Remission	439 (36)
No recovery	40 (3.3)
Death	8 (0.7)
After-effects	125 (10.3)
Unknown	2 (0.2)

**Figure 1:**
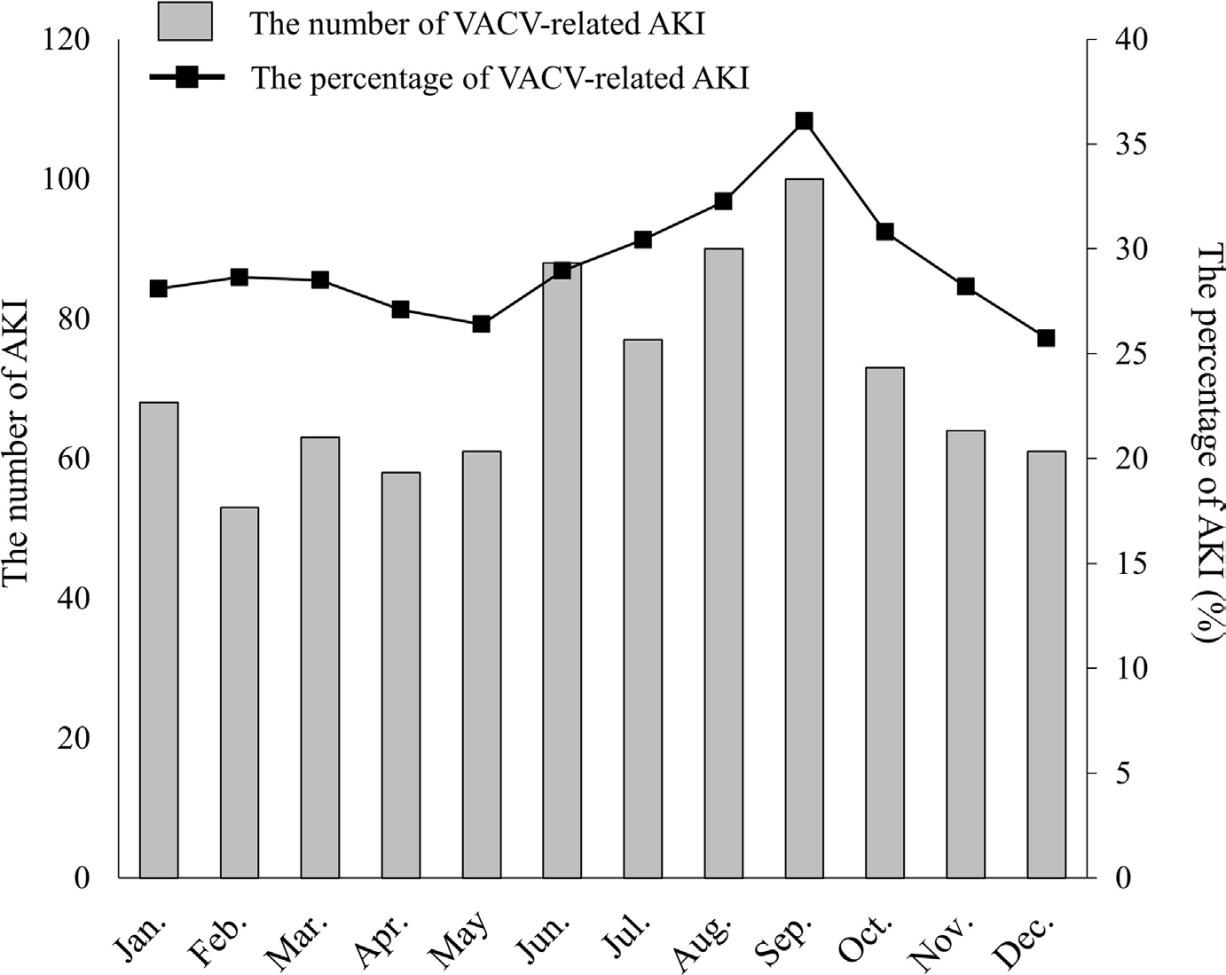
Seasonal distribution of VACV-related AKI. The number of VACV-related AKI cases by month is indicated as shaded columns. The percentage of AKI cases versus all VACV-related adverse events is indicated by the line graph (solid line).

To examine the seasonal distribution of VACV-related AKI, we extracted 2,907 VACV-related ADE occurrence cases between January 2005 and December 2017. (Some cases in a given reported year did not always experience an ADE in the same year in JADER dataset. That is, the reported year did not necessarily correspond to the year in which ADE developed. To consider seasonality appropriately, we evaluated 2,097 VACV-related ADE occurrence cases instead of the reported cases in this period.) The number and percentage of VACV-related AKI events are shown in **Figure 1**. The number of VACV-related AKI events increased between June and September, and the same trend was observed for the percentage of AKI events. However, this seasonal variation was not observed for AKI cases associated with cisplatin use (**Supplementary Figure 1**).

### Signal Comparison of AKI With Concomitant Use of VACV and Analgesics

A total of 437,738 cases (including the 1,212 VACV-related AKI cases) were included in the analysis. The signals of VACV-related AKI were compared using the ROR. As shown in **Table 2**, the ROR for cases with concomitant use of VACV and NSAIDs was significantly higher than that for the cases with VACV monotherapy (adjusted ROR: 13.60 [12.52–14.77] vs 28.76 [24.76–33.41], *p* < 0.0001). In contrast, the ROR for cases with concomitant use of VACV and acetaminophen was not higher than that for VACV monotherapy. Similar trends in PRR and EBGM were also observed (**Supplementary Table 2**).

**Table 2:** Comparison of AKI signals with concomitant use of analgesics.

	Cases with AKI	Cases without AKI	Crude ROR (95% CI)	Adjusted ROR (95% CI)	*p* value
**Valacyclovir**	864	2,020	14.11 (13.00–15.31)	13.60 (12.52–14.77)	<0.0001
**NSAIDs**	1,588	43,091	1.22 (1.15–1.28)	1.29 (1.22–1.36)	<0.0001
**Acetaminophen**	284	7,654	1.22 (1.09–1.38)	1.23 (1.09–1.38)	0.0008
**VACV and NSAIDs**	328	390	27.74 (23.92–32.16)	28.76 (24.76–33.41)	<0.0001
**VACV and acetaminophen**	20	123	5.36 (3.34–8.61)	5.22 (3.24–8.39)	<0.0001
**Reference group**	11,223	370,153	1	1	–

### Characteristics of AKI With Concomitant Use of VACV and NSAIDs

**Figure 2:**
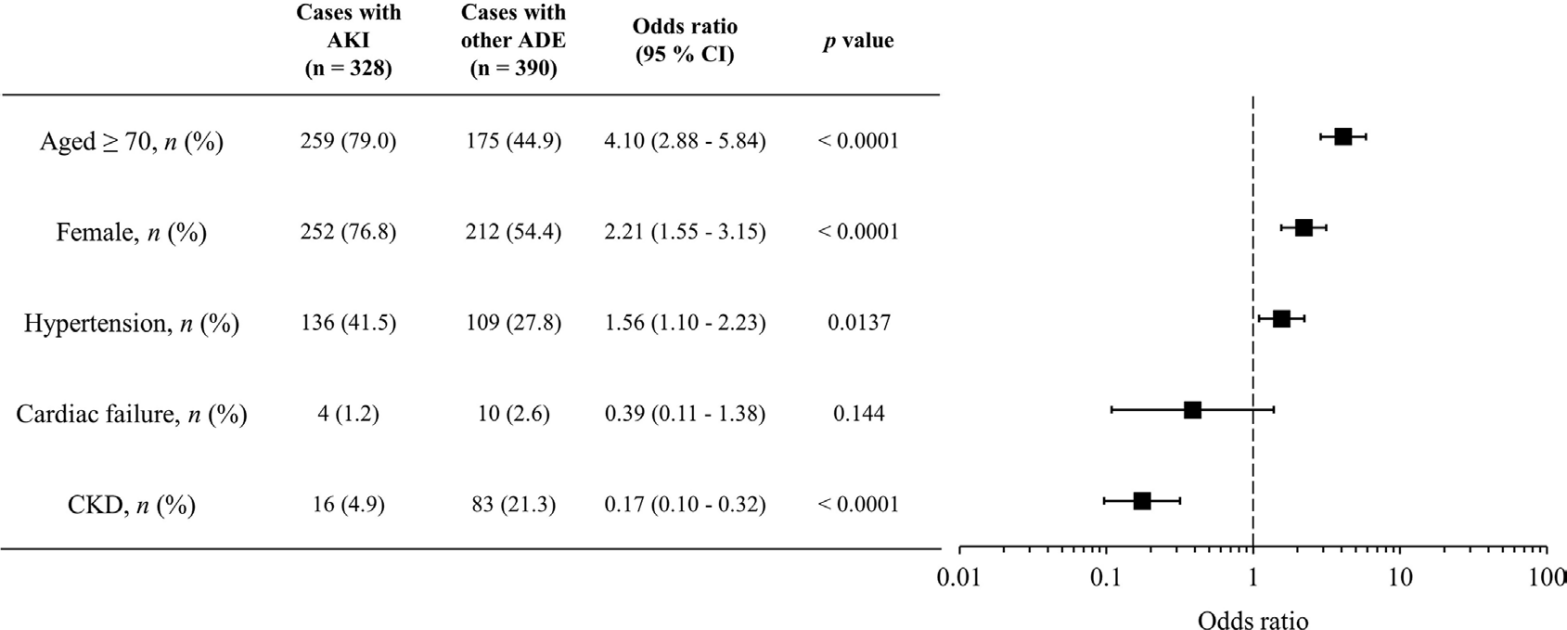
Characteristics of candidate risk factors for AKI with concomitant use of VACV and NSAIDs. Boxes represent odds ratio, and vertical lines represent 95% confidence interval.

In total, 718 ADE cases with concomitant use of VACV and NSAIDs were reported, of which 328 VACV-related AKI cases were observed. From the multivariate logistic regression analysis, patients with AKI were more likely to be older (≥70 years) (odds ratio [OR]: 4.10 [2.88–5.84], *p* < 0.0001), to be female (OR: 2.21 [1.55–3.15], *p* < 0.0001), or to have hypertension (OR: 1.56 [1.10–2.23], *p* < 0.0001) (**Figure 2**). Diabetes mellitus was not associated with VACV-related AKI in the cases with concomitant use of NSAIDs.

### Signal Evaluation of VACV-Related AKI With Concomitant Use of RAS Inhibitors and NSAIDs

**Figure 3:**
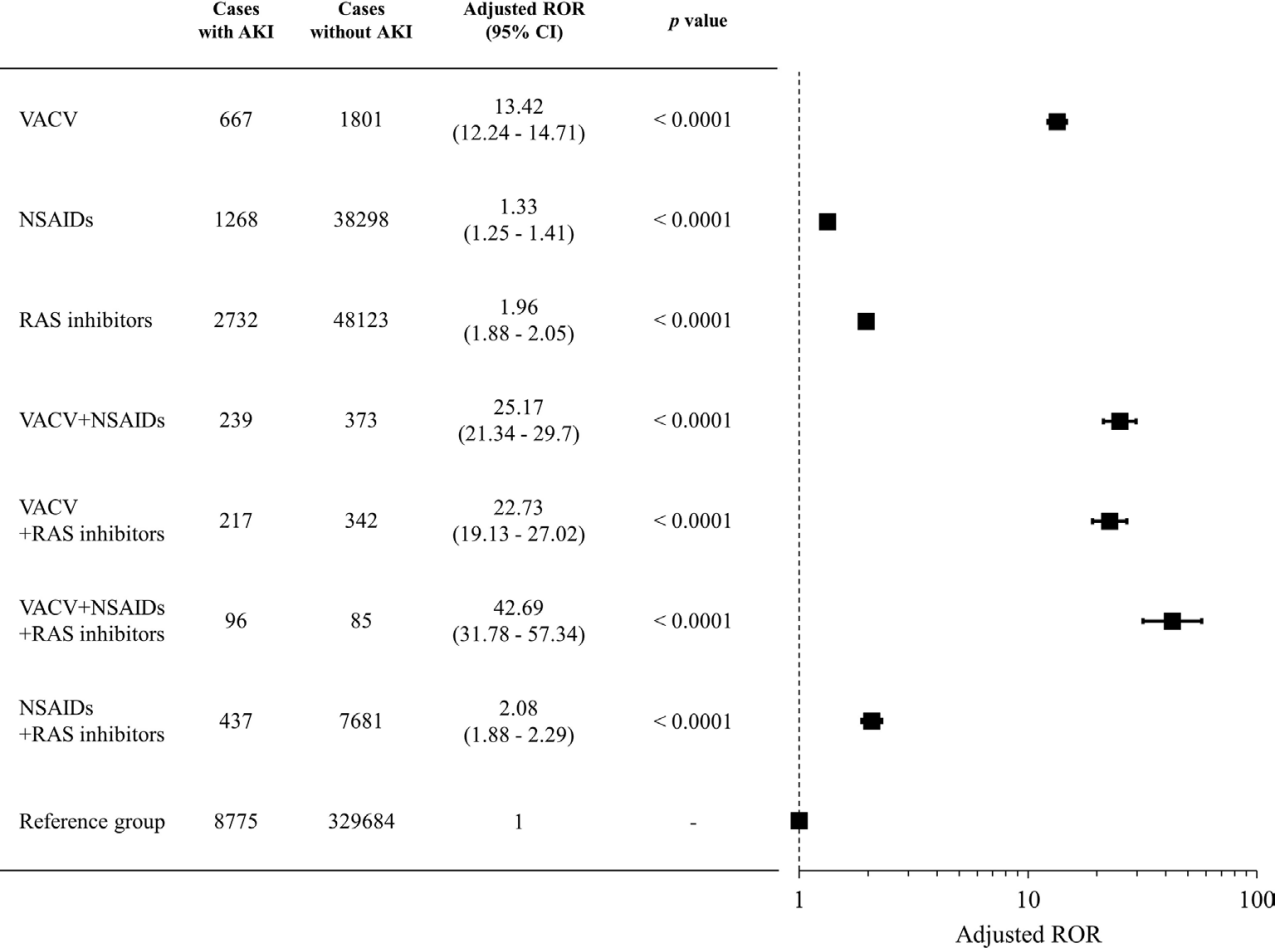
Signals of VACV-related AKI with concomitant use of RAS inhibitors and NSAIDs. Boxes represent reporting odds ratio (ROR), and vertical lines represent 95% confidence interval. RORs were adjusted for age, sex, and reporting year.

We evaluated the signals of VACV-related AKI with concomitant use of RAS inhibitors and/or NSAIDs. The RORs for each group are shown in **Figure 3**. As with the cases with concomitant use of NSAIDs, cases with concomitant use of VACV and RAS inhibitors had a significantly higher ROR than cases with monotherapy of either drug type (*p* < 0.0001). Furthermore, the ROR in the cases with triple concomitant use (VACV and RAS inhibitors and NSAIDs) was significantly higher than that of any other group (*p* < 0.0001). A similar trend was observed for the other parameters (**Supplementary Table 3**).

## Discussion

In the present analysis, we demonstrated a signal increase for AKI in cases with concomitant use of VACV and NSAIDs, while no signal increase was observed in cases with concomitant use of VACV and acetaminophen. Additionally, our findings showed that patients who were older, were female, or had hypertension were at higher risk of AKI with concomitant use of VACV and NSAIDs. Furthermore, we demonstrated that concomitant use of RAS inhibitors and NSAIDs increased the signal of VACV-related AKI.

Drug-induced AKI is associated with multiple drug types, and AKI is a common event in clinical practice (Schetz et al., 2005; Pannu and Nadim, 2008; Pazhayattil and Shirali, 2014). In the JADER database, VACV is the most commonly reported drug suspected of causing AKI (Hosohata et al., 2019), indicating the importance of identifying the population at high risk for VACV-related AKI. In our study, many of the VACV-related AKI cases were female and older in age (**Table 1**). Older age and male sex have been identified as risk factors for the frequency and severity of AKI from previous experimental and clinical studies (Chertow et al., 1998; Xue et al., 2006; Coca, 2010; Aufhauser et al., 2016). Contrary to these findings, however, the number of female patients with VACV-related AKI was greater than the number of male patients in our study. A previous population-based study demonstrated that both VACV and ACV, the active form of VACV, were more commonly used by women than by men (Sawyer et al., 1988). This observation may explain why there were more female than male patients with VACV-related AKI in our study.

Interestingly, an increase in the number and percentage of VACV-related AKI cases was observed in summer in Japan (June to September) (**Figure 1**). Dehydration plays an important role in ACV-induced AKI (Vachvanichsanong et al., 1995; Yildiz et al., 2012). Most regions in Japan have distinct seasonal variations, and summer is the most common season for dehydration and heat shock to be reported (Takinami and Maeda, 2016). Additionally, several studies have reported that Japanese patients with hypertension showed lower renal function in summer (Masugata et al., 2011; Imai and Abe, 2013). Dehydration is also known as a risk factor for various types of AKI other than ACV-induced AKI. However, although dehydration is a risk factor for cisplatin-induced AKI, no seasonal variation was observed for AKI cases with cisplatin in JADER (**Supplementary Figure 1**). In Japan, almost all chemotherapy with standard-dose cisplatin is administered on an inpatient basis with intravenous hydration (Okazaki et al., 2013), while VACV is typically prescribed to outpatients without hydration, and this may have affected the differences of seasonal distribution observed between VACV and cisplatin. Taken together, these findings indicate the importance of increased clinical awareness of VACV-related AKI in the summer months.

Analgesics are essential drugs for pain management of herpes zoster and also to reduce the risk of postherpetic neuralgia (Jeon, 2015). Therefore, AKI risk evaluation for analgesics in concomitant use with VACV is needed. In previous studies, Yue et al. (2014), Yue et al. (2018) found that concomitant use of VACV and NSAIDs might increase AKI risk. However, the risk of AKI with concomitant use of acetaminophen remains unclear. In the present study, we verified that the ROR for cases with concomitant use of VACV and NSAIDs was significantly higher than that for cases with VACV monotherapy in JADER. We also determined that a signal elevation of AKI was not detected in cases with concomitant use of VACV and acetaminophen (**Table 2**). Additionally, the AKI signal in the FDA Adverse Event Reporting System (FAERS) was in accordance with the signal in JADER (**Supplementary Table 4**). However, the ROR for cases with concomitant use of VACV and acetaminophen was significantly lower than that for cases with VACV monotherapy in JADER. Kadowaki et al. (2012) reported that acetaminophen attenuates oxidative stress and protects the remaining kidney function in an adenine-induced rat model of chronic renal failure. Therefore, we examined data from FAERS to determine whether acetaminophen had a preventive effect against VACV-related AKI *via* a drug–drug interaction, but no such interaction between acetaminophen and VACV was observed (**Supplementary Table 4**). These results suggest that acetaminophen does not at least increase the risk of VACV-related AKI. Although further studies are needed to clarify whether acetaminophen exerts a renal protective effect, we considered acetaminophen to be safer than NSAIDs with regard to VACV-related AKI.

To prevent AKI caused by drug–drug interactions between NSAIDs and VACV, it is necessary to identify the high-risk populations for VACV-related AKI with concomitant use of NSAIDs. Thus, we evaluated the relationships between patient characteristics and occurrence of VACV-related AKI in cases with concomitant use of NSAIDs by multivariate analysis and found that older age (≥70 years), female sex, and presence of hypertension were related to AKI with concomitant use of VACV and NSAIDs (**Figure 2**). These factors have been previously reported as risk factors for drug-induced renal impairment (Pazhayattil and Shirali, 2014). However, in cases with concomitant use of VACV and acetaminophen, no relationship between these risk factors and the occurrence of AKI was observed (**Supplementary Table 5**), indicating that risk factors for VACV-related AKI may vary according to the type of concomitant analgesic. Although further studies are needed, these findings indicate that acetaminophen may be a more appropriate analgesic than NSAIDs in high-risk populations such as patients who are older, are female, and with hypertension.

RAS inhibitors are among the most commonly prescribed classes of drug for hypertension. Although RAS inhibitors can slow the progression of CKD, they can lead to acute prerenal failure *via* a similar mechanism to that of NSAIDs. Therefore, we evaluated the signal of VACV-related AKI with concomitant use of RAS inhibitors. The ROR for AKI with concomitant use of VACV and RAS inhibitors was significantly higher than that for VACV monotherapy, similar to the concomitant use of NSAIDs (**Figure 3**). Interestingly, a further increase in the ROR for AKI was observed with combined use of VACV and both NSAIDs and RAS inhibitors (**Figure 3**). These findings suggest that concomitant use of VACV and NSAIDs should be carefully considered, particularly in patients already receiving RAS inhibitors.

The mechanism of drug–drug interaction between VACV and NSAIDs and/or RAS inhibitors has not been proved. Yue et al. (2018) proposed that an increase in plasma levels of VACV can lead to increased occurrence of AKI *via* inhibition of the organic anion transporters by NSAIDs. In the present study, we focused on the relationship between decreased urinary output and occurrence of VACV-related AKI. Several studies have reported that VACV- and ACV-induced AKI is associated with the production of insoluble crystals in the distal tubular lumen (Sawyer et al., 1988; Mason and Nickols, 2008; Roberts et al., 2011), indicating that a decrease in urinary output may be a risk factor for VACV-related AKI (Perazella, 1999). We observed an increase in VACV-related AKI signals with concomitant use of RAS inhibitors as well as of NSAIDs. These drugs can decrease urinary output *via* a hemodynamic reduction in glomerular filtration by arteriole constriction (Schoolwerth Anton et al., 2001; Dreischulte et al., 2015). In contrast, previous studies have suggested that acetaminophen does not affect renal function under clinical use conditions (Capuzzo et al., 1999; Farquhar et al., 1999). Indeed, in cases with concomitant use of acetaminophen, no increase in AKI signal was observed in our study. These results do not contradict our hypothesis. Additionally, we investigated the signal of VACV-related AKI with concomitant use of furosemide and found an association with decreased rather than increased signal (**Supplementary Table 6**). Furosemide can lead to increasing urine output, which may prevent VACV-induced postrenal kidney injury by suppressing insoluble crystal production. Furthermore, multiple VACV-related AKI cases were observed in summer. These results also support our hypothesis that urinary output loss plays an important role in VACV-related AKI. On the other hand, the concomitant use of nephrotoxic agents such as cisplatin and gentamycin did not increase the signal of VACV-related AKI (data not shown).

Based on these results, the clinical implication of the present study is the need for greater attention to decreased urinary output caused by concomitant drugs and/or environmental factors when prescribing VACV. Furthermore, acetaminophen may represent an appropriate analgesic for patients at high risk of VACV-related AKI.

This study has some limitations. First, spontaneous reporting systems (SRSs), such as the JADER database, have a reporting bias because cases are reported voluntarily and under-reporting may therefore occur. Moreover, the true number of cases involving each drug is unclear in SRS databases; therefore, the incidence of adverse events could not be calculated in our study. Second, we determined the concomitant use of analgesics and RAS inhibitors from the SRS database, and cases of AKI with concomitant use of these drugs might be overlooked because of a lack of information about the concomitant use of drugs, meaning that drug–drug interactions attributed to NSAIDs and RAS inhibitors might have been underestimated in this study. Third, SRS databases can lack information on clinical details such as drug use (Almenoff et al., 2005). The JADER dataset lacks clinical information such as laboratory test data (serum creatinine, blood urea nitrogen, and cystatin C), and cases have many missing values for height, weight, and dose and frequency of drug administration. Therefore, we could not evaluate the renal function and dose of VACV and concomitant drugs in each case. Fourth, the number of cases in the JADER database was lower than that in the FAERS because only adverse events reported in Japan are contained in the JADER database. This lower number of cases might have affected the signal detection of AKI. Despite this limitation, the JADER database has more information than the FAERS on each case, including data on concomitant drug use, duration of medication, and comorbidities (Okada et al., 2019). Therefore, we used the JADER database to identify risk factors for AKI with concomitant use of VACV and NSAIDs. To address these limitations, further investigation, including a population-based study, is required. Finally, it is not possible to fully determine the extent to which reduced GFR contributes to the risk of VACV-induced (or ACV-induced) AKI in an epidemiological or observational study. Further studies are therefore required to clarify the mechanism of drug–drug interactions between VACV and NSAIDs and/or RAS inhibitors.

## Conclusions

In the present study, we demonstrated that many VACV-related AKI cases occur in summer in Japan. Our findings also suggest that acetaminophen might represent a safer analgesic than NSAIDs with respect to VACV-related AKI. Additionally, we identified older age, female sex, and hypertension as candidate risk factors for VACV-related AKI with concomitant use of NSAIDs. Furthermore, our findings suggest that concomitant use of RAS inhibitors is related to VACV-related AKI, with or without the use of NSAIDs. Although further studies are warranted, our findings suggest that it is necessary to consider the particular concomitant drug used and the seasonal factor for the prevention of VACV-related AKI.

## Data Availability

The datasets generated for this study are available on request to the corresponding author.

## Author Contributions

II and YK designed this study. II, YK SI, ST, KM, KO, JS, and AA analyzed the data. YK, YI, and TI drafted the manuscript. All authors have read and approved the final manuscript.

## Funding

This work was supported by JSPS KAKENHI (grant numbers JP17K15748 and JP19K13914).

## Conflict of Interest Statement

The authors declare that the research was conducted in the absence of any commercial or financial relationships that could be construed as a potential conflict of interest.
