# Supplementary figures and images for: Risk Evaluation for Acute Kidney Injury Induced by the Concomitant Use of Valacyclovir, Analgesics, and Renin–Angiotensin System Inhibitors: The Detection of Signals of Drug–Drug Interactions
Source: Front Pharmacol. 2019 Aug 8;10:874. doi: 10.3389/fphar.2019.00874 (PMC6694181; doi:10.3389/fphar.2019.00874)

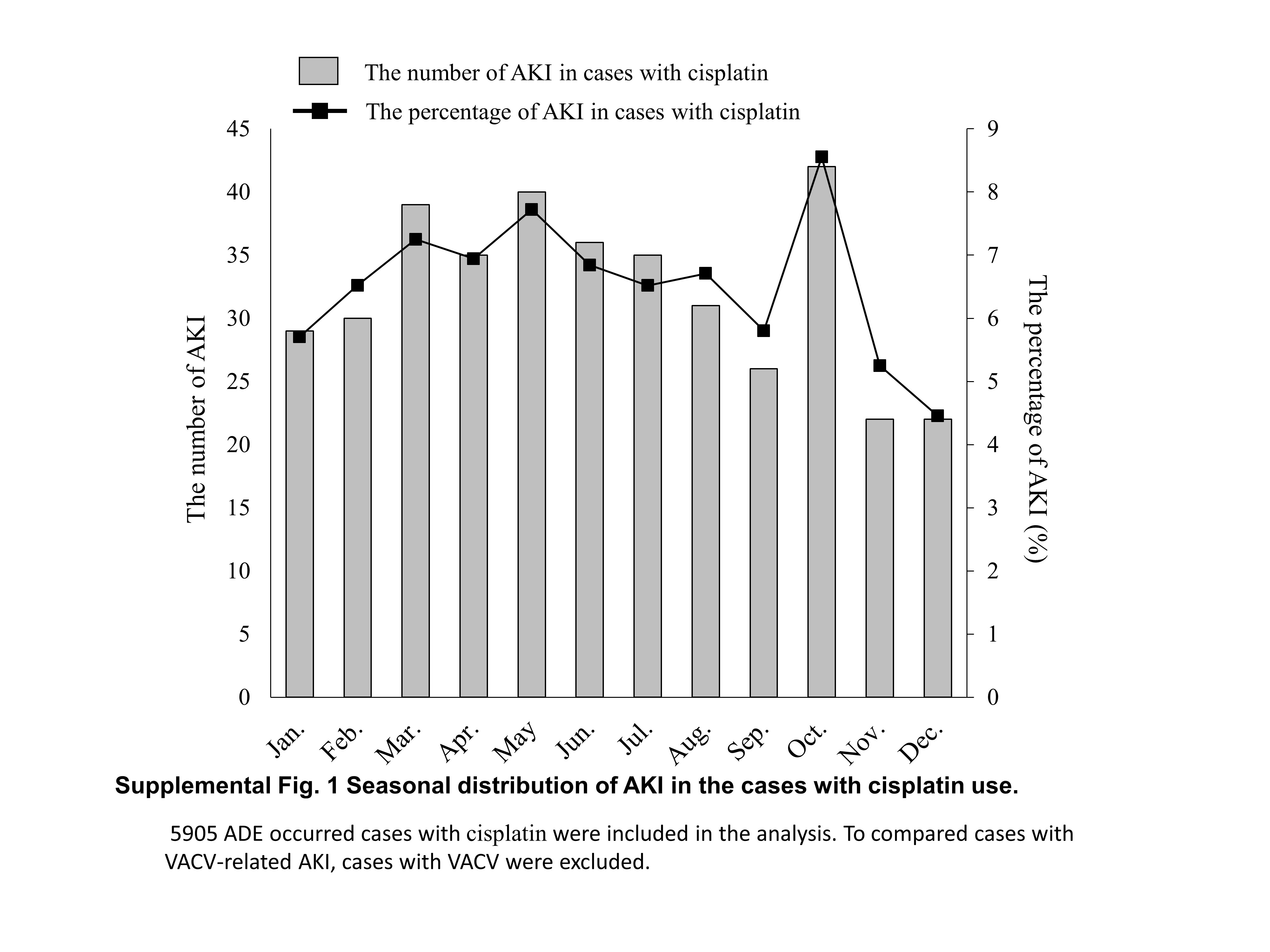

Supplement: Supplementary file 1 [file Image_1.jpeg]
