# Supplementary material for: Risk Evaluation for Acute Kidney Injury Induced by the Concomitant Use of Valacyclovir, Analgesics, and Renin–Angiotensin System Inhibitors: The Detection of Signals of Drug–Drug Interactions
Source: Front Pharmacol. 2019 Aug 8;10:874. doi: 10.3389/fphar.2019.00874 (PMC6694181; doi:10.3389/fphar.2019.00874)
Supplement: Supplementary file 2 [file Table_1.docx]

Supplementary Table 1a PT list for identification of acute kidney injury events

| Standard MedDRA Query | PT code | PT |
| --- | --- | --- |
| “Acute renal failure” [20000003] | 10069339 | Acute kidney injury |
|  | 10069688 | Acute phosphate nephropathy |
|  | 10002847 | Anuria |
|  | 10003885 | Azotaemia |
|  | 10066338 | Continuous haemodiafiltration |
|  | 10061105 | Dialysis |
|  | 10078987 | Foetal renal impairment |
|  | 10018875 | Haemodialysis |
|  | 10053090 | Haemofiltration |
|  | 10049778 | Neonatal anuria |
|  | 10029155 | Nephropathy toxic |
|  | 10030302 | Oliguria |
|  | 10034660 | Peritoneal dialysis |
|  | 10072370 | Prerenal failure |
|  | 10038435 | Renal failure |
|  | 10038447 | Renal failure neonatal |
|  | 10062237 | Renal impairment |
|  | 10049776 | Renal impairment neonatal |

Supplementary Table 1b PT list used for identification of chronic kidney disease events

| Standard MedDRA Query | PT code | PT |
| --- | --- | --- |
| “Chronic kidney disease” [20000213] | 10053699 | Artificial kidney device user |
|  | 10003885 | Azotaemia |
|  | 10064848 | Chronic kidney disease |
|  | 10078095 | Chronic kidney disease-mineral and bone disorder |
|  | 10010082 | Coma uraemic |
|  | 10012660 | Diabetic end stage renal disease |
|  | 10061105 | Dialysis |
|  | 10059015 | Dialysis device insertion |
|  | 10077512 | End stage renal disease |
|  | 10018367 | Glomerulonephritis chronic |
|  | 10018875 | Haemodialysis |
|  | 10053090 | Haemofiltration |
|  | 10019845 | Hepatorenal failure |
|  | 10062624 | High turnover osteopathy |
|  | 10020708 | Hyperparathyroidism secondary |
|  | 10023421 | Kidney fibrosis |
|  | 10063000 | Low turnover osteopathy |
|  | 10081588 | Metabolic nephropathy |
|  | 10058116 | Nephrogenic anaemia |
|  | 10067467 | Nephrogenic systemic fibrosis |
|  | 10029159 | Nephrosclerosis |
|  | 10049630 | Oedema due to renal disease |
|  | 10034498 | Pericarditis uraemic |
|  | 10034660 | Peritoneal dialysis |
|  | 10052279 | Renal and liver transplant |
|  | 10052278 | Renal and pancreas transplant |
|  | 10038435 | Renal failure |
|  | 10074746 | Renal replacement therapy |
|  | 10038519 | Renal rickets |
|  | 10038533 | Renal transplant |
|  | 10056609 | Uraemia odour |
|  | 10046324 | Uraemic acidosis |
|  | 10046326 | Uraemic encephalopathy |
|  | 10063709 | Uraemic gastropathy |
|  | 10077910 | Uraemic myopathy |
|  | 10046328 | Uraemic neuropathy |
|  | 10060875 | Uraemic pruritus |
|  | 10067863 | Uridrosis |
|  | 10070869 | Acquired cystic kidney disease |
|  | 10069688 | Acute phosphate nephropathy |
|  | 10001582 | Albumin urine present |
|  | 10001580 | Albuminuria |
|  | 10072735 | Aluminium overload |
|  | 10077087 | Autoimmune nephritis |
|  | 10004783 | Biopsy kidney abnormal |
|  | 10053811 | Blood 1,25-dihydroxycholecalciferol decreased |
|  | 10005358 | Blood bicarbonate abnormal |
|  | 10005359 | Blood bicarbonate decreased |
|  | 10005393 | Blood calcium abnormal |
|  | 10005395 | Blood calcium decreased |
|  | 10005481 | Blood creatinine abnormal |
|  | 10005483 | Blood creatinine increased |
|  | 10050671 | Blood erythropoietin abnormal |
|  | 10053796 | Blood erythropoietin decreased |
|  | 10005700 | Blood parathyroid hormone abnormal |
|  | 10005703 | Blood parathyroid hormone increased |
|  | 10054823 | Blood phosphorus abnormal |
|  | 10050196 | Blood phosphorus increased |
|  | 10005722 | Blood potassium abnormal |
|  | 10005725 | Blood potassium increased |
|  | 10005800 | Blood sodium abnormal |
|  | 10005802 | Blood sodium decreased |
|  | 10005846 | Blood urea abnormal |
|  | 10005851 | Blood urea increased |
|  | 10067442 | Bloody peritoneal effluent |
|  | 10005952 | Bone cyst |
|  | 10081461 | C1q nephropathy |
|  | 10077827 | C3 glomerulopathy |
|  | 10049797 | Calcification of muscle |
|  | 10051714 | Calciphylaxis |
|  | 10063209 | Chronic allograft nephropathy |
|  | 10068447 | Creatinine renal clearance abnormal |
|  | 10011372 | Creatinine renal clearance decreased |
|  | 10078114 | Destructive spondyloarthropathy |
|  | 10061835 | Diabetic nephropathy |
|  | 10064553 | Dialysis amyloidosis |
|  | 10059256 | Dialysis disequilibrium syndrome |
|  | 10076665 | Dialysis membrane reaction |
|  | 10071946 | Dialysis related complication |
|  | 10054832 | Diffuse mesangial sclerosis |
|  | 10068883 | Effective peritoneal surface area increased |
|  | 10014625 | Encephalopathy |
|  | 10058363 | Eosinophils urine present |
|  | 10068800 | Extensive interdialytic weight gain |
|  | 10068279 | Fibrillary glomerulonephritis |
|  | 10067757 | Focal segmental glomerulosclerosis |
|  | 10018356 | Glomerular filtration rate abnormal |
|  | 10018358 | Glomerular filtration rate decreased |
|  | 10018364 | Glomerulonephritis |
|  | 10018370 | Glomerulonephritis membranoproliferative |
|  | 10018372 | Glomerulonephritis membranous |
|  | 10018374 | Glomerulonephritis minimal lesion |
|  | 10018376 | Glomerulonephritis proliferative |
|  | 10018378 | Glomerulonephritis rapidly progressive |
|  | 10051920 | Glomerulonephropathy |
|  | 10061989 | Glomerulosclerosis |
|  | 10018620 | Goodpasture's syndrome |
|  | 10070737 | HIV associated nephropathy |
|  | 10070476 | Haemodialysis complication |
|  | 10059268 | Haemodialysis-induced symptom |
|  | 10018932 | Haemolytic uraemic syndrome |
|  | 10062713 | Haemorrhagic diathesis |
|  | 10075015 | Haemorrhagic fever with renal syndrome |
|  | 10068610 | Hepatitis virus-associated nephropathy |
|  | 10020586 | Hypercalcaemic nephropathy |
|  | 10062747 | Hypercreatininaemia |
|  | 10020646 | Hyperkalaemia |
|  | 10020705 | Hyperparathyroidism |
|  | 10020711 | Hyperphosphataemia |
|  | 10055171 | Hypertensive nephropathy |
|  | 10020919 | Hypervolaemia |
|  | 10020942 | Hypoalbuminaemia |
|  | 10020947 | Hypocalcaemia |
|  | 10021036 | Hyponatraemia |
|  | 10021263 | IgA nephropathy |
|  | 10077209 | IgM nephropathy |
|  | 10067871 | Immunotactoid glomerulonephritis |
|  | 10077108 | Inadequate haemodialysis |
|  | 10022530 | Intercapillary glomerulosclerosis |
|  | 10074739 | Intradialytic parenteral nutrition |
|  | 10022870 | Inulin renal clearance decreased |
|  | 10069384 | Ischaemic nephropathy |
|  | 10023435 | Kidney small |
|  | 10050791 | Leukocyturia |
|  | 10025140 | Lupus nephritis |
|  | 10066453 | Mesangioproliferative glomerulonephritis |
|  | 10027417 | Metabolic acidosis |
|  | 10027525 | Microalbuminuria |
|  | 10065673 | Nephritic syndrome |
|  | 10029151 | Nephropathy |
|  | 10029155 | Nephropathy toxic |
|  | 10029164 | Nephrotic syndrome |
|  | 10029782 | Normochromic anaemia |
|  | 10029783 | Normochromic normocytic anaemia |
|  | 10029784 | Normocytic anaemia |
|  | 10077862 | Obstructive nephropathy |
|  | 10031250 | Osteomalacia |
|  | 10076749 | Paraneoplastic glomerulonephritis |
|  | 10075626 | Paraneoplastic nephrotic syndrome |
|  | 10051653 | Parathyroid gland enlargement |
|  | 10034484 | Pericarditis |
|  | 10067011 | Peritoneal cloudy effluent |
|  | 10067594 | Peritoneal dialysis complication |
|  | 10069638 | Peritoneal effluent abnormal |
|  | 10067301 | Peritoneal effluent erythrocyte count increased |
|  | 10067300 | Peritoneal effluent leukocyte count increased |
|  | 10072490 | Peritoneal equilibration test abnormal |
|  | 10059524 | Peritoneal fluid analysis abnormal |
|  | 10069000 | Peritoneal fluid protein abnormal |
|  | 10068998 | Peritoneal fluid protein increased |
|  | 10070442 | Peritoneal permeability increased |
|  | 10062622 | Pigment nephropathy |
|  | 10065381 | Polyomavirus-associated nephropathy |
|  | 10075849 | Potassium wasting nephropathy |
|  | 10053123 | Protein urine present |
|  | 10037032 | Proteinuria |
|  | 10038182 | Red blood cells urine positive |
|  | 10065427 | Reflux nephropathy |
|  | 10038357 | Renal amyloidosis |
|  | 10038381 | Renal atrophy |
|  | 10038491 | Renal papillary necrosis |
|  | 10038536 | Renal tubular atrophy |
|  | 10039834 | Secondary hypertension |
|  | 10048302 | Tubulointerstitial nephritis |
|  | 10069568 | Ultrafiltration failure |
|  | 10045422 | Ultrasound kidney abnormal |
|  | 10046337 | Urate nephropathy |
|  | 10046358 | Urea renal clearance decreased |
|  | 10067534 | Urinary casts present |
|  | 10053544 | Urine albumin |
|  | 10053541 | Urine albumin |
|  | 10059895 | Urine output decreased |
|  | 10053539 | Urine protein |
|  | 10053538 | Urine protein |
|  | 10051753 | Vascular calcification |
|  | 10047967 | White blood cells urine positive |

Supplementary Table 1c PT list used for identification of hypertension

| Standard MedDRA Query | PT code | PT |
| --- | --- | --- |
| “Hypertension” [20000147] | 10000358 | Accelerated hypertension |
|  | 10005732 | Blood pressure ambulatory increased |
|  | 10005739 | Blood pressure diastolic increased |
|  | 10051128 | Blood pressure inadequately controlled |
|  | 10005750 | Blood pressure increased |
|  | 10063926 | Blood pressure management |
|  | 10053355 | Blood pressure orthostatic increased |
|  | 10005760 | Blood pressure systolic increased |
|  | 10081751 | Catecholamine crisis |
|  | 10012758 | Diastolic hypertension |
|  | 10014129 | Eclampsia |
|  | 10057615 | Endocrine hypertension |
|  | 10015488 | Essential hypertension |
|  | 10070538 | Gestational hypertension |
|  | 10049058 | HELLP syndrome |
|  | 10020571 | Hyperaldosteronism |
|  | 10020772 | Hypertension |
|  | 10049781 | Hypertension neonatal |
|  | 10059238 | Hypertensive angiopathy |
|  | 10020801 | Hypertensive cardiomegaly |
|  | 10058222 | Hypertensive cardiomyopathy |
|  | 10077000 | Hypertensive cerebrovascular disease |
|  | 10020802 | Hypertensive crisis |
|  | 10058179 | Hypertensive emergency |
|  | 10020803 | Hypertensive encephalopathy |
|  | 10079496 | Hypertensive end-organ damage |
|  | 10020823 | Hypertensive heart disease |
|  | 10055171 | Hypertensive nephropathy |
|  | 10049079 | Labile hypertension |
|  | 10025600 | Malignant hypertension |
|  | 10025603 | Malignant hypertensive heart disease |
|  | 10026674 | Malignant renal hypertension |
|  | 10026924 | Maternal hypertension affecting foetus |
|  | 10026985 | Mean arterial pressure increased |
|  | 10052066 | Metabolic syndrome |
|  | 10067598 | Neurogenic hypertension |
|  | 10065508 | Orthostatic hypertension |
|  | 10076704 | Page kidney |
|  | 10036485 | Pre-eclampsia |
|  | 10065918 | Prehypertension |
|  | 10062886 | Procedural hypertension |
|  | 10038464 | Renal hypertension |
|  | 10074864 | Renal sympathetic nerve ablation |
|  | 10038562 | Renovascular hypertension |
|  | 10038926 | Retinopathy hypertensive |
|  | 10039808 | Secondary aldosteronism |
|  | 10039834 | Secondary hypertension |
|  | 10078932 | Supine hypertension |
|  | 10042957 | Systolic hypertension |
|  | 10048007 | Withdrawal hypertension |

Supplementary Table 1d PT list used for identification of diabetes mellitus

| Standard MedDRA Query | PT code | PT |
| --- | --- | --- |
| “Hyperglycaemia/ new onset diabetes mellitus” [20000041] | 10073667 | Acquired lipoatrophic diabetes |
|  | 10065367 | Blood 1,5-anhydroglucitol decreased |
|  | 10005557 | Blood glucose increased |
|  | 10012596 | Diabetes complicating pregnancy |
|  | 10012601 | Diabetes mellitus |
|  | 10012607 | Diabetes mellitus inadequate control |
|  | 10012631 | Diabetes with hyperosmolarity |
|  | 10077357 | Diabetic arteritis |
|  | 10012650 | Diabetic coma |
|  | 10080788 | Diabetic coronary microangiopathy |
|  | 10071265 | Diabetic hepatopathy |
|  | 10012668 | Diabetic hyperglycaemic coma |
|  | 10012669 | Diabetic hyperosmolar coma |
|  | 10012671 | Diabetic ketoacidosis |
|  | 10012672 | Diabetic ketoacidotic hyperglycaemic coma |
|  | 10012673 | Diabetic ketosis |
|  | 10074309 | Diabetic metabolic decompensation |
|  | 10081558 | Diabetic wound |
|  | 10080061 | Euglycaemic diabetic ketoacidosis |
|  | 10017395 | Fructosamine increased |
|  | 10072628 | Fulminant type 1 diabetes mellitus |
|  | 10018209 | Gestational diabetes |
|  | 10018429 | Glucose tolerance impaired |
|  | 10018430 | Glucose tolerance impaired in pregnancy |
|  | 10018478 | Glucose urine present |
|  | 10018473 | Glycosuria |
|  | 10018475 | Glycosuria during pregnancy |
|  | 10018481 | Glycosylated haemoglobin abnormal |
|  | 10018484 | Glycosylated haemoglobin increased |
|  | 10020635 | Hyperglycaemia |
|  | 10063554 | Hyperglycaemic hyperosmolar nonketotic syndrome |
|  | 10071394 | Hyperglycaemic seizure |
|  | 10071286 | Hyperglycaemic unconsciousness |
|  | 10056997 | Impaired fasting glucose |
|  | 10022489 | Insulin resistance |
|  | 10022490 | Insulin resistance syndrome |
|  | 10022491 | Insulin resistant diabetes |
|  | 10053247 | Insulin-requiring type 2 diabetes mellitus |
|  | 10023379 | Ketoacidosis |
|  | 10023388 | Ketonuria |
|  | 10023391 | Ketosis |
|  | 10023392 | Ketosis-prone diabetes mellitus |
|  | 10066389 | Latent autoimmune diabetes in adults |
|  | 10052066 | Metabolic syndrome |
|  | 10075980 | Monogenic diabetes |
|  | 10028933 | Neonatal diabetes mellitus |
|  | 10033660 | Pancreatogenous diabetes |
|  | 10081755 | Steroid diabetes |
|  | 10067584 | Type 1 diabetes mellitus |
|  | 10067585 | Type 2 diabetes mellitus |
|  | 10072659 | Type 3 diabetes mellitus |
|  | 10057597 | Urine ketone body present |

Supplementary Table 1e PT list used for identification of cardiac failure

| Standard MedDRA Query | PT code | PT |
| --- | --- | --- |
| “Cardiac failure” [2000000420000147] | 10063081 | Acute left ventricular failure |
|  | 10001029 | Acute pulmonary oedema |
|  | 10063082 | Acute right ventricular failure |
|  | 10007522 | Cardiac asthma |
|  | 10007554 | Cardiac failure |
|  | 10007556 | Cardiac failure acute |
|  | 10007558 | Cardiac failure chronic |
|  | 10007559 | Cardiac failure congestive |
|  | 10007560 | Cardiac failure high output |
|  | 10007625 | Cardiogenic shock |
|  | 10051093 | Cardiopulmonary failure |
|  | 10068230 | Cardiorenal syndrome |
|  | 10063083 | Chronic left ventricular failure |
|  | 10063084 | Chronic right ventricular failure |
|  | 10010968 | Cor pulmonale |
|  | 10010969 | Cor pulmonale acute |
|  | 10010970 | Cor pulmonale chronic |
|  | 10050528 | Ejection fraction decreased |
|  | 10019645 | Hepatic congestion |
|  | 10051448 | Hepatojugular reflux |
|  | 10024119 | Left ventricular failure |
|  | 10024899 | Low cardiac output syndrome |
|  | 10049780 | Neonatal cardiac failure |
|  | 10073708 | Obstructive shock |
|  | 10037423 | Pulmonary oedema |
|  | 10050459 | Pulmonary oedema neonatal |
|  | 10076203 | Radiation associated cardiac failure |
|  | 10075337 | Right ventricular ejection fraction decreased |
|  | 10039163 | Right ventricular failure |
|  | 10060953 | Ventricular failure |

**Supplementary Table 2 Comparison of AKI signals with concomitant use of analgesics using PRR and EBGM**

|  | **Cases  with AKI** | **Cases  without AKI** | **PRR** | **χ2** | **EBGM** | **EB05** |
| --- | --- | --- | --- | --- | --- | --- |
| **Valacyclovir** | 864 | 2,020 | 10.18 | 6848.24 | 9.75 | 9.14 |
| **NSAIDs** | 1,588 | 43,091 | 1.21 | 51.06 | 1.10 | 1.05 |
| **Acetaminophen** | 284 | 7,654 | 1.22 | 10.71 | 1.05 | 0.94 |
| **VACV and NSAIDs** | 328 | 390 | 15.52 | 4450.75 | 14.67 | 13.23 |
| **VACV and acetaminophen** | 20 | 123 | 4.75 | 57.15 | 3.89 | 1.00 |

PRR, proportional reporting ratios; EBGM, empirical Bayes geometric mean; EB05, lower limit of the 95% confidence interval; VACV, valacyclovir; NSAIDs, non-steroidal anti-inflammatory drugs

**Supplementary Table 3 VACV-related AKI signals with concomitant use of RAS inhibitors and NSAIDs using PRR and EBGM**

|  | **Cases  with AKI** | **Cases  without AKI** | **PRR** | **χ2** | **EBGM** | **EB05** |
| --- | --- | --- | --- | --- | --- | --- |
| **VACV** | 667 | 1,801 | 10.42 | 5422.80 | 8.57 | 8.04 |
| **NSAIDs** | 1,268 | 38,298 | 1.24 | 51.09 | 0.97 | 0.93 |
| **RAS inhibitors** | 2,732 | 48,123 | 2.07 | 1189.84 | 1.63 | 1.58 |
| **VACV+NSAIDs** | 239 | 373 | 15.06 | 3124.01 | 13.06 | 11.77 |
| **VACV+RAS inhibitors** | 217 | 342 | 14.97 | 2822.56 | 12.14 | 10.87 |
| **VACV+NSAIDs+RAS inhibitors** | 96 | 85 | 20.46 | 1784.94 | 15.62 | 13.34 |
| **NSAIDs+RAS inhibitors** | 437 | 7,681 | 2.08 | 237.52 | 1.63 | 1.50 |

**Supplementary Table 4 Comparison with VACV-related AKI signal in FAERS**

|  | **Cases  with AKI** | **Cases  without AKI** | **Crude ROR  (95% CI)** | **Adjusted ROR  (95% CI)** | ***p* value** |
| --- | --- | --- | --- | --- | --- |
| **Valacyclovir** | 617 | 7,868 | 3.12  (2.87–3.39) | 3.65  (3.36–3.96) | < 0.0001 |
| **NSAIDs** | 2,780 | 76,105 | 1.58  (1.52–1.64) | 1.70  (1.63–1.77) | < 0.0001 |
| **Acetaminophen** | 3,649 | 76,735 | 2.09  (2.02–2.16) | 2.21  (2.14–2.29) | < 0.0001 |
| **VACV and NSAIDs** | 182 | 719 | 9.55  (1.52–1.64) | 11.77  (10.00–13.85) | < 0.0001 |
| **VACV and acetaminophen** | 51 | 715 | 3.15  (2.36–4.19) | 3.32  (2.50–4.41) | < 0.0001 |
| **Reference group** | 94,815 | 4,409,350 | 1 | 1 | - |

**Supplementary Table 5**

**Characteristics of candidate risk factors for AKI with concomitant use of VACV and acetaminophen**

|  | **Cases with AKI (n=20)** | **Cases without AKI (n)** | **Odds ratio (95% CI)** | ***p* value** |
| --- | --- | --- | --- | --- |
| Age ≥70, n (%) | 13 (65.0) | 69 (56.1) | 2.16  (0.79–5.86) | 0.132 |
| Hypertension, *n* (%) | 8 (40.0) | 27 (22.0) | 2.11  (0.77–5.79) | 0.147 |

To characterize the risk factors for AKI with concomitant use of VACV and acetaminophen, a case-control study of the cohort was conducted. The method used in this analysis was the same as that for concomitant use of VACV and NSAIDs. The explanatory variables in the final model were selected using a stepwise method according to *p* value. The final model is shown in Supplemental Table 5.

**Supplementary Table 6 VACV-related AKI signals with concomitant use of furosemide**

|  | **Cases  with AKI** | **Cases  without AKI** | **Crude ROR  (95% CI)** | **Adjusted ROR  (95% CI)** | ***p* value** |
| --- | --- | --- | --- | --- | --- |
| **Valacyclovir** | 1,173 | 2,344 | 17.53 (16.31–18.85) | 17.14 (15.93–18.44) | < 0.0001 |
| **Furosemide** | 1,777 | 23,166 | 2.69 (2.55–2.83) | 2.50 (2.37–2.63) | < 0.0001 |
| **Valacyclovir and furosemide** | 46 | 257 | 6.27 (4.58–8.59) | 6.05 (4.41–8.29) | < 0.0001 |
| **Reference group** | 11,435 | 400,620 | 1 | 1 | - |
